# Complex structural variations in non-human primate hepatitis B virus

**DOI:** 10.1186/s12985-021-01667-0

**Published:** 2021-10-09

**Authors:** Yoshihito Nagura, Kei Fujiwara, Kentaro Matsuura, Etsuko Iio, Yasuhito Tanaka, Hiromi Kataoka

**Affiliations:** 1 Department of Gastroenterology and Metabolism, Nagoya City University Graduate School of Medical Sciences, 1 Kawasumi, Mizuho, Mizuho, Nagoya, Aichi 467-8601 Japan; 2 Department of Virology and Liver Unit, Nagoya City University Graduate School of Medicinal Sciences, Nagoya, 467-8601 Japan

**Keywords:** Hepatitis B virus, Non-human primate, Complex structural variation

## Abstract

**Background:**

Recent genome sequence technology has revealed a novel type of genetic rearrangement referred to as complex structural variations (SVs). Previous studies have elucidated the complex SVs in human hepatitis B viruses (HBVs). In this study, we investigated the existence of complex SVs in HBVs from non-human primates (NHPs).

**Methods:**

Searches for nucleotide sequences of NHP HBV were conducted using the PubMed, and genetic sequences were retrieved from databases. The candidate genetic sequences harboring complex SVs were analyzed using the CLUSTALW program and MAFFT. Additional bioinformatical analyses were performed to determine strains with complex SVs and to elucidate characteristics of NHP HBV strains.

**Results:**

One hundred and fifty-four HBV strains from NHPs were identified from databases. SVs and complex SVs were observed in 11 (7.1%) strains. Three gibbon HBV (GiHBV) strains showed complex SVs consisting of an insertion and a deletion in the pre-S1 region. One GiHBV strain possessed a 6-nt insertion, which are normally specific to human HBV genotype A (HBV/A) in the Core region, and further analyses clarified that the 6-nt insertion was not caused by recombination, but rather by simple insertion. Another chimpanzee HBV strain showed complex SVs in the pre-S1 region, which were composed of human HBV/E, G-specific polymorphic SV, and an additional 6-nt insertion.

**Conclusions:**

In this study, complex SVs were observed in HBV strains from NHPs, in addition to human HBV strains, as shown in previous studies. These data suggest that complex SVs could also be found in other members of hepadnaviruses, and may play a role in their genetic diversity.

**Supplementary Information:**

The online version contains supplementary material available at 10.1186/s12985-021-01667-0.

## Background

More than 257 million people worldwide are infected with chronic hepatitis B virus (HBV), and 20% to 30% of people with untreated chronic HBV infection progress to liver cirrhosis, which may lead to liver failure and hepatocellular carcinoma (HCC) [1]. A total of 0.5 to 1.2 million deaths per year are caused by HBV infection, mainly due to cirrhosis and HCC, the latter accounting for 320,000 deaths annually [2]. Presently, HBV infection is one of the serious public health problems globally such as malaria or human immunodeficiency virus.

HBV genetic alterations are important virologically and clinically. Among the genetic changes observed in the HBV genome, point mutations such as pre-core mutation or core promoter mutations have been reported, and these mutations affect the pathogenesis of HBV-related liver disease [3–6]. In addition, recombinations are reported to be important genetic changes [7–9].

Human HBV is a prototype of the family *Hepadnaviradae*, which are subdivided into 2 genera: *Orthohepadnavirus* which infects mammals, and *Avihepadnavirus* which infects birds [10]. In addition, recent studies have revealed novel hepadnaviruses in amphibians [11], and fish [12]. Research on these non-human hepadnaviruses is very important. For example, experiment of initial steps of hepadnavirus infection was performed by duck hepatitis B virus originally, which later led to the discovery of sodium/taurocholate cotransporting polypeptide (NTCP) receptor which is very important factor in HBV infection [13, 14].

Recent genome sequence technology has revealed a novel type of genetic rearrangement, referred as complex structural variations (SVs). Complex SVs are defined by genetic sequences composed of multiple breakpoints whose origin cannot be explained by a single end-joining or DNA exchange event [15], and practically, are formed by 2 or more SVs co-occurring at the same locus [16]. Initially using a unique HBV strain with an unreported rearrangement [17], we identified a novel non-canonical form of genetic change, referred to as complex SVs, in human HBV [18, 19]. Furthermore, we found polymorphic SVs in human HBV genotypes A to H and in HBVs from non-human primates (NHPs) in the pre-S1 region [18]. In addition, recent research revealed that polymorphic SVs were observed among various orthohepadnaviruses [20]. Based on the previous studies reporting various types of complex SVs in human HBV, this study aimed to clarify the existence of complex SVs in HBVs from NHPs.

## Methods

### HBV genetic sequence search

Searches for nucleotide sequences of NHP HBV were conducted using the PubMed, and genetic sequences were retrieved from DDBJ/EMBL/Genbank. In addition, research articles analyzing nucleotide sequences of NHP HBV were identified in the PubMed database.

### Reference sequence

V00866 (HBV/A) was used as a reference sequence. In addition, the consensus sequences of human HBV/A, HBV/B, HBV/C, HBV/D, HBV/E, HBV/F, HBV/G, HBV/H and HBV/I were determined using the CLUSTALW program [21] by analyzing 150, 31, 168, 78, 37, 38, 13, 30 and 3 complete genome sequences, respectively. In addition, consensus sequences of each non-human primate HBV were also determined using CLUSTALW.

### Analysis of simple and complex SVs

Simple canonical SVs were determined by multiple alignment of the reference sequence of HBV/A (V00866). Complex SVs are defined by the existence of SVs with multiple breakpoints and are composed of a complex mixture of deletions, insertions, and duplications [15, 16]. The candidate genetic sequences harboring complex SVs were analyzed using the CLUSTALW program and MAFFT [22], and alignments with the reference sequence (HBV/A, V00866) were determined. When the sequences contained a portion with low sequence similarity to the reference sequences with and without gaps, the partial genetic sequences were analyzed using NCBI BLAST 2.2.31 [23]. Then additional manual inspection considering the result of NCBI BLAST analysis was conducted, and complex SVs were determined based on the patterns of complex SVs in previously published articles as references [18, 19].

### Phylogenetic analysis and recombination analysis

Phylogenetic analysis with neighbor-joining methods was performed using MEGA software version 6 [24]. Bootstrapping resampling and reconstruction with 1000 replicates were performed. Genetic distance calculation and pairwise distance comparison were performed using Kimura’s two-parameter model integrated into the MEGA software. SIMPLOT program version 3.5.1[25] was used to analyze inter-genotypic recombination of HBV strains.

### Percent identity analysis

Percent identities among HBV strains from human genotypes A to I, and NHPs, were analyzed by MAFFT [22].

## Results

### HBV strains from NHPs

HBV genetic sequences from NHPs were searched in databases and published articles. One hundred and fifty-four HBV genetic sequences from NHPs (109 complete genome sequences and 45 partial genome sequences) were retrieved. Of these, 91, 41, 16, and 6 were from gibbon HBV (GiHBV), chimpanzee HBV (ChHBV), orangutan HBV (OuHBV), and gorilla HBV (GoHBV), respectively. More than half of the genetic sequences were from GiHBV. Detailed information on the NHP HBV genetic sequences are shown in Additional file 1. Phylogenetic analysis using 63 complete genome sequences from human and NHP HBV strains demonstrated that human and NHP HBV strains showed distinct groups (Additional file 1: Figure S1). Multiple alignment analysis was performed using MAFFT [22], and all the NHP HBV strains were compared with the reference sequence (V00866, human HBV/A, or AF046996, Woolly monkey HBV (WMHBV)) and consensus sequences of human HBV genotypes A to H, and HBV from NHPs. For complex SVs, 70 HBV strains with complex SVs described in the previous reports [18, 19] were considered as prototypes for searching for complex SVs.

### SVs in HBV strains from NHPs

**Fig. 1:**
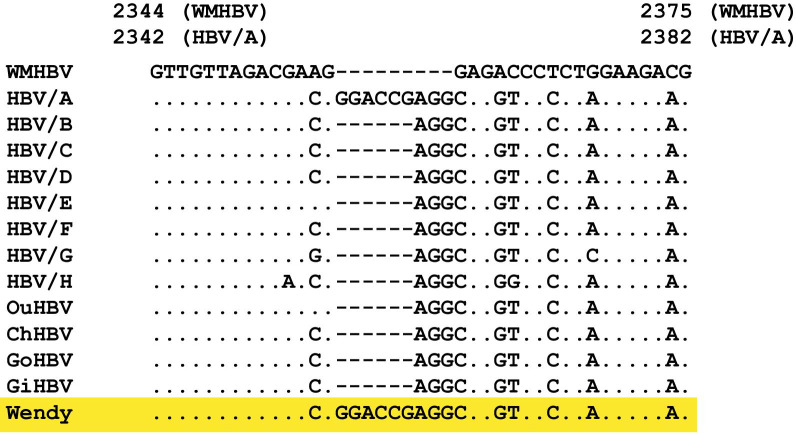
Six nucleotide (nt) insertion specific to hepatitis B virus genotype A (HBV/A) was observed in Wendy strain. The consensus genetic sequences of human HBV/A to H, and HBVs from non-human primates (NHPs) in the Core region were aligned with the reference sequence of woolly monkey HBV (WMHBV). Compared with human HBV/B to H and HBVs from NHPs, HBV/A has polymorphic 6-nt insertion in the Core region. One gibbon HBV strain (Wendy) highlighted in yellow also possessed the same 6-nt insertion as HBV/A in the Core region

**Fig. 2:**
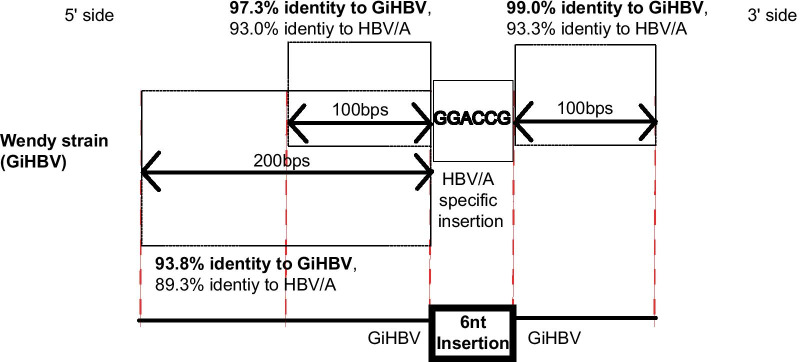
Analysis of percent identities in both the 5ʹ and 3ʹ side of the 6-nt insertion in the Core region of Wendy strain. Detailed results of the percent identity analyses are shown in Additional file 1: Tables S1–S3. GiHBV, gibbon hepatitis B virus (HBV); HBV/A, HBV genotype A

SVs were observed in 11 strains (7.1%) in HBV strains from NHPs (Table 1). Canonical simple SVs were observed in 7 HBV strains (4.5%) from NHPs. Of these, 4 (2.6%) were deletions and 3 (1.9%) were insertions, respectively. Interestingly, 1 GiHBV strain (Wendy, AY330914) showed 6-nt insertion in the Core region, which was generally observed only in human HBV/A (Fig. 1). All the other HBV strains from NHPs did not possess the 6-nt insertion (Table 1). Percent identity analyses by MAFFT indicated that the Wendy strain did not show high genetic sequences identities to HBV/A strains in both the 5ʹ (100 and 200 bps) and 3ʹ (100 bps) sides of the 6-nt insertion, which indicated that the 6-nt insertion in Wendy strain was not caused by recombination between GiHBV and HBV/A (Fig. 2, Additional file 1: Tables S1–S3). Thus, Wendy strain showed higher genetic percent identities to GiHBV in both the 5ʹ and 3ʹ side of the 6-nt insertion (Fig. 2, Additional file 1: Tables S1–S3), suggesting that the HBV/A specific nucleotide fragment of 6-nt observed in Wendy strain was an insertion.

**Table 1:** SVs and complex SVs in HBV from non-human primates

	SV		6nt insertion of HBV/A in the Core region	Pre-S1 pattern (non-human primate type)
	Simple	Complex		
*Orangutan (n* = *16)*				
Yes	0	0	0	10
No	16	16	10	0
NA	0	0	6	6
*Gorilla (n* = *6)*				
Yes	0	0	0	6
No	6	6	6	0
NA	0	0	0	0
*Chimp (n* = *41)*				
Yes	3	1	0	30
No	38	40	27	**1**
NA	0	0	14	10
*Gibbon (n* = *91)*				
Yes	4	3	1	67
No	87	88	69	0
NA	0	0	21	24

SV, structural variation; HBV/A, hepatitis B virus genotype A; NA, not applicable

The existence of 6-nt insertion in the Core region in all the human HBV strains used in the determination of consensus sequences (150 HBV/A, 31 HBV/B, 168 HBV/C, 78 HBV/D, 37 HBV/E, 38 HBV/F, 13 HBV/G, 30 HBV/H, and 3 HBV/I) were analyzed. One HBV/A strain lacked the 6-nt insertion. The HBV strain was further analyzed. The strain showed HBV/A sequence in the 5ʹ side and 3ʹ side of the missing 6-nt region. It was speculated that 6-nt insertion was deleted in this strain. Therefore, 6-nt insertion in the Core region was conserved in 151/152 (99.3%) of human HBV/A strains. Wendy strain was the only one strain (1/445 (0.2%)) that possessed 6-nt insertion in the Core region among HBV/B to I and HBV from NHPs.

### Complex SVs in HBV strains from NHPs

**Fig. 3:**
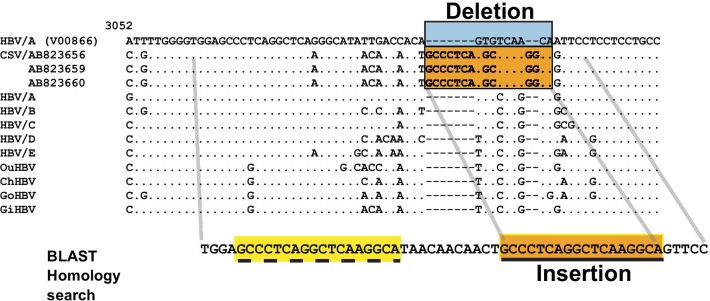
Complex structural variations (SVs) observed in 3 gibbon hepatitis B virus (GiHBV) strains in the pre-S1 region. The genetic sequences of 3 GiHBV strains were aligned with the reference sequence of V00866, consensus sequences of human HBV/A to E, and non-human primate HBVs. The segment underlined and highlighted in orange was searched in NCBI BLAST, and the results showed that the segment was identical to the segment just to the 5ʹ side of the searched segment shown as the part highlighted in yellow with the dotted underline. In addition, the segment surrounded by the light blue rectangle was deleted. Therefore, complex SVs were composed of insertion and deletion

Three GiHBV strains showed sequence gaps with the reference sequence in the pre-S1 region. The sequence fragments with gaps in the GiHBVs were analyzed using NCBI-BLAST [23]. The result of the BLAST search showed that the fragments were insertions from the 5ʹ side of the area. Therefore, the rearrangement was consisted of deletion and insertion, as shown in Fig. 3.

**Fig. 4:**
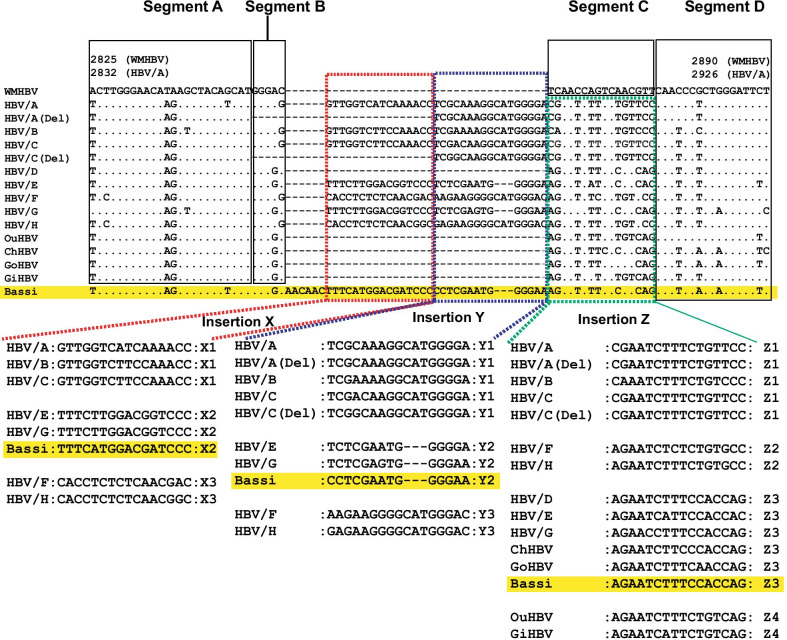
Polymorphic structural variations (SVs) in human and non-human primate (NHP) HBVs in the pre-S1 region, and complex SVs observed in Bassi strain. Unique polymorphic SVs observed in human and NHP HBVs were analyzed in a previous study [18] and multiple alignment was created using woolly monkey HBV (WMHBV) as a reference. In addition, Bassi strain specific complex SVs observed in the pre-S1 region were shown. Segment A, B, and D, are conserved regions. Insertions X, Y, and Z are polymorphic SVs, and are further divided into X1-X3, Y1-Y3, and Z1-Z4, respectively [18]. (Del) shows deletion pattern minor variants (deletion of segment B and insertion X) observed only in human HBV/A and HBV/C. Bassi strain was highlighted in yellow. HBV/A, hepatitis B virus genotype A; Del, deletion; OuHBV, orangutan HBV; ChHBV, chimpanzee HBV, GoHBV, gorilla HBV; GiHBV, gibbon HBV

In a previous study [18], polymorphic SVs were discovered in the pre-S1 region among human HBV genotypes A to H, and NHP HBVs. Briefly, the rearrangements were composed of insertions of unknown origin (X, Y, Z) and deletion of segment C of WMHBV as shown in Fig. 4. Thus, insertions X, Y and Z were classified as X1 to X3, Y1 to Y3, and Z1 to Z4 according to percent identities of nucleotide sequences less than 80.0%. Some HBV/A, C strains showed deletion of X and segment B, described as HBV/A (Del) and HBV/C (Del). Human HBV/D and non-human primate HBVs did not possess insertion X and Y. For insertion Z, HBV/D, E, G, ChHBV and GoHBV showed a similar pattern (Z3), and OuHBV and GiHBV showed a different pattern (Z4). Comprehensively, HBV/A to C, HBV/E and G, HBV/F and H showed similar patterns; X1 + Y1 + Z1, X2 + Y2 + Z3, and X3 + Y3 + Z2, respectively [18]. These variations were basically conserved in all the human HBVs and NHP HBVs. However, 1 ChHBV strain (Bassi, AB046525) showed complex SVs in pre-S1 region. As shown in Fig. 4, human HBV/D and NHP HBVs possess a deletion type pattern in the X + Y region. However, Bassi strain showed an insertion X2 + Y2 pattern, which was characteristics of human HBV/E and G. In the Z region, Bassi showed a common pattern of human HBV/D, E, G, ChHBV, and GoHBV. Furthermore, Bassi strain contained the Bassi-specific 6-nt insertion “AACAAC” in the 5ʹ side of the insertion X region, as shown in Fig. 4. This insertion was not observed in other human nor NHP HBVs. Therefore, Bassi sequence in this region was composed of 2 different insertions: X2 + Y2, which was characteristics of human HBV/E, G, and the other was “AACAAC”. No human HBV sequences showed a similar pattern.

**Fig. 5:**
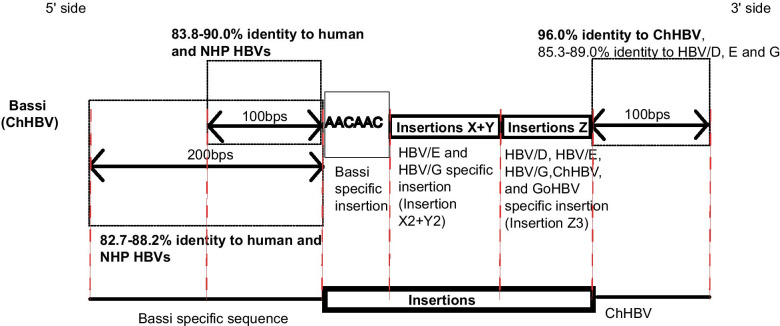
Analysis of percent identities in both the 5ʹ and 3ʹ side of complex SVs in Bassi strain. ChHBV, chimpanzee hepatitis B virus; HBV/D, HBV genotype D; NHP, non-human primate. Detailed percent identity analyses are shown in Additional file 1: Tables S4–S6

Percent identity analysis of 5ʹ side of this complex SVs (100 and 200 bps) showed that Bassi had the Bassi-specific sequence, indicating that none of the human nor NHP HBV strains showed high genetic percent identities to Bassi strain (Fig. 5, and Additional file 1: Tables S4–S6). No clear evidence of recombination with human HBV genotypes A-I or NHP HBV was observed in the recombination analysis. In addition, percent identity analysis of the 3ʹ side of this complex SVs (100 bps) showed that Bassi strain belonged to ChHBV (Fig. 5, and Additional file 1: Tables S4–S6). In the above-mentioned pre-S1 area, each HBV genotypes and NHP HBVs possessed specific polymorphic SVs/complex SVs, and furthermore, the Bassi strain showed its specific complex SVs, which were composed of 2 insertions. Bassi strain had a chimpanzee compatible genetic sequence in the 3ʹ side from the complex SV site, and Bassi strain had its own specific genetic sequences in the 5ʹ side from the complex SV site.

## Discussion

Complex SVs were reported in human and mouse genome analysis initially [15, 16]. Complex SVs in HBV were first reported in human HBV, and furthermore, unique polymorphic SVs were observed in the pre-S1 region of human and NHP HBVs [18–20]. In this study, SVs and complex SVs in NHP HBV were searched and clarified. Three GiHBV strains showed complex SVs composed of insertion and deletion in the pre-S1 region (nt 3093–3102 of the reference strain (V00866)). In the previous human HBV analysis, more than 90% of complex SVs were observed in nt 1500–2000 which contained X and Core regions [19]. The position and the insertional motif of the complex SVs in the GiHBVs were not similar to those observed in human HBV. The pattern of complex SVs, insertion and deletion, was the most frequent pattern of complex SVs observed in human HBV.

Our analysis showed that 6-nt insertion observed in Wendy strain was not caused by recombination, but rather by SV. Both the 5ʹ and 3ʹ sides of the 6-nt insertion in Wendy did not show high sequence identities with human HBV/A, and they showed high sequence identities with GiHBVs. Similarly, both the 5ʹ and 3ʹ sides of the pre-S1 insertions of Bassi did not show high sequence identities with HBV/E or HBV/G; therefore, the pre-S1 region of Bassi was complex SVs composed of 2 insertions. It is not clear why the SV and complex SVs in Wendy and Bassi occurred; however, it is speculated that 2 regions (6-nt insertion in the Core region in HBV/A, and the pre-S1 region where polymorphic SVs were observed in HBVs) were unique regions where nucleotide breakpoints could occur. As for the Bassi-specific sequence in the 5ʹ side of the complex SV site in the pre-S1 region, unique partial divergence of Bassi strain from other human and NHP HBVs in the Core region was previously reported [26]. This study clarified that Bassi strain had complex SVs in the 3ʹ end of the partial unique Bassi-specific genetic sequence.

Polymorphic SVs in the pre-S1 region are very unique structures. Previous studies reported resemblance of HBV/D and NHP HBVs, as well as resemblance of HBV/E and HBV/G in this region [27, 28]. A previous study [18] further clarified the genetic structure of the pre-S1 region. These polymorphic SVs caused the differences in the lengths of human and NHP HBVs. The differences in length of human HBV genotypes and NHP HBVs (3215 nt length of human HBV/ B, C, F, H, I; 3212 nt length of human HBV/E; 3182 nt length of human HBV/D and NHP HBVs) are caused by the polymorphic SVs in the pre-S1 region. In addition, 6-nt insertion in the Core region along with the pre-S1 structure (common to HBV/A, B, C) determined 3221 nt length of human HBV/A, and 36 nt insertion in the Core region along with the pre-S1 structure (common to HBV/E and G) determined 3248 nt length of human HBV/G. SVs in HBV have not been extensively studied; however, our study along with previous studies [18, 20] have elucidated their importance. The SVs appeared as both polymorphic changes, which were observed in certain HBV genotypes, and sporadic changes, which were observed in 3 GiHBVs, Bassi and Wendy. These SVs also implicate that genome breakpoints occurred both polymorphically and sporadically.

Cross-species transmission between human HBV and NHP HBVs has been proposed as the origin of HBV, among other theories [10]. Polymorphisms of the pre-S1 SVs suggest a possible rationale. As described, the polymorphic pre-S1 SVs were highly conserved. Pre-S1 deletion type was conserved in NHP HBVs globally. In humans, the pre-S1 deletion type was observed only in HBV/D. For example, in Indonesia, HBV/B and HBV/C are distributed as human HBV genotypes [29]; both of HBV/B and C possess insertions X and Y in the pre-S1 region, and the pre-S1 X + Y deletion type was prevalent in HBV from NHP in Indonesia. This suggest that the direct transmission of NHP HBV to human HBV in Indonesia is unlikely. Further, contact between NHP HBV in Indonesia and geographically distant human HBV/D is unlikely. A recent study reported that 2 HBV strains recovered from neolithic human skeletons (7000 and 5000 years ago) were most closely related to HBV from African NHP, and 1 HBV strain recovered from a medieval human skeleton (3000 years ago) belonged to HBV/D [30]. This study showed compatible data with our result. If cross-species transmission occurred, the first human HBV should be similar to HBV from NHP, and HBV/D would be the second human HBV, considering the common polymorphic lack of insertion X and Y in the pre-S1 region. In addition, complex SVs in the pre-S1 region observed in Bassi strain may suggest the origin of polymorphic diversity in human HBV. Polymorphic SVs observed in human HBV/A-C, E and G, F and H might have been inserted in the X and Y deleted prototype, as observed in Bassi strain, and have been maintained thereafter. These explanations may not be sufficient to clarify the origin of HBV at this time. Remaining unanswered questions may be clarified incrementally as data on the genetic sequences of orthohepadnavirus are further accumulated.

As described in the previous article, the complex SVs can rearrange genome to create novel proteins, shuffle promoters or enhancers into a novel regulatory configuration [15]. Experimental studies of complex SVs in human HBV strains have shown that complex SVs with hepatocyte nuclear factor 1 (HNF1) binding site in basic core promoter in HBV caused accumulation of Hepatitis B core protein in nucleus and perinucleus [17], and in addition, same phenomenon was observed in liver pathology of a patient infected with HBV strain which contained complex SVs. Experimental data also showed that the construct with complex SVs containing HNF1 binding site showed higher pregenomic and preS/S RNA levels [17, 31]. Complex SVs can modulate HBV pathobiology by affecting transcription and protein production. Further studies may clarify the role of complex SVs in HBV.

## Conclusion

Previous reports have shown that novel complex SVs are observed in human HBV [18, 19]. This study clarified that SVs and complex SVs are also observed in NHP HBVs. This findings suggest that complex SVs could be found in other members of hepadnaviruses. Further studies are required to clarify the impact of complex SVs on the virological characteristics and genetic diversity of the viruses.

## Supplementary Information

**Additional file 1.** List of human HBV sequences. List of non-human primate HBVs. **Table S1.** Percent identities among Wendy, human HBV/A-I, and non-human primate HBVs in 100 bp of 5’ side of the 6nt insertion specific to HBV/A in the Core region. **Table S2.** Percent identities among Wendy, human HBV/A-I, and non-human primate HBVs in 200 bp of 5’ side of the 6nt insertion specific to HBV/A in the Core region. **Table S3.** Percent identities among Wendy, human HBV/A-I, and non-human primate HBVs in 100 bp of 3’ side of the 6nt insertion specific to HBV/A in the Core region. **Table S4.** Percent identities among Bassi, human HBV/A-I, and nonhuman primate HBVs in 100 bp of 5’ side of the polymorphic preS1 region. **Table S5.** Percent identities among Bassi, human HBV/A-I, and non-human primate HBVs in 200 bp of 5’ side of the polymorphic preS1 region. **Table S6.** Percent identities among Bassi, human HBV/A-I, and non-human primate HBVs in 100 bp of 3’ side of the polymorphic preS1 region. **Figure S1.** Phylogenetic analysis of hepatitis B virus (HBV) was performed by the neighbor-joining method for human and non-human primate (NHP) HBVs.

## Data Availability

The datasets used and/or analyzed during the current study are available from the corresponding author on reasonable request.

## Abbreviations

SV: Structural variation
HBV: Hepatitis B virus
NHP: Non-human primate
GiHBV: Gibbon HBV
ChHBV: Chimpanzee HBV
OuHBV: Orangutan HBV
GoHBV: Gorilla HBV
