# Supplementary material for: Complex structural variations in non-human primate hepatitis B virus
Source: Virol J. 2021 Oct 9;18:200. doi: 10.1186/s12985-021-01667-0 (PMC8501659; doi:10.1186/s12985-021-01667-0)

**Supplementary information**

**List of human HBV sequences**

HBV/A (n=150)

A1 (n=69): FM199977, GU563548, FM199979, HM535205, DQ020002, AF297621, AF297622, AY233290, AY233284, AY233281, AY233288, AY233283, AY233285, FJ692585, HE974363, AB241114, AB116091, AB453986, AB453987, AB241115, AB116088, AB076679, AB116093, AY903452, AB453989, AB453988, AY934771, AY373429, KJ638661, AY934768, AY934767, AY934769, AY934770, AY934772, AY934766, AB116089, AY233274, AF297625, AB116082, AB116087, M57663, AB076678, AB116094, AB116084, AB116086, DQ020003, EF103278, AY161141, AY373432, AF297623, AY233275, AY233276, AY233277, AY233278, AY233279, AY233282, AY233287, AY233289, AF418674, AY161140, AY161138, AB116083, U87742, EU366129, AB116085, FM199974, AY934773, AM494718, AB246335

A2 (n=56): AB205118, AY233280, AF297624, S50225, V00866, Z35717, X70185, EU594392, EU594390, EU594391, EU594395, EU594394, EU594384, EU594386, EU594383, EU594393,

EU594388, EU594389, EU594387, EU594385, AY152726, AY902775, AB116077, AJ012207, AB116078, Z72478, AB116080, L13994, AB116079, X02763, GU563546, AB116081, AB014370,

AJ344115, AF090838, X51970, AF090841, AB116076, EU859911, EU859929, GU563554, EU859942, X70185, AB064314, AF090839, AF090840, AF536524, AF537371, AF537372, AJ309369, AJ309370, AJ309371, AY034878, AY128092, GQ477461, EU859948

A3 (n=6): AB194952, AB194951, AB194950, AM184126, AM180623, AM184125

A4 (n=1): AY934764

A5 (n=5): FJ692613, FJ692611, FJ692554, FJ692556, FN545831

A6 (n=3): GQ331047, GQ331046, GQ331048

A7 (n=10): FN545830, FN545840, FN545839, FN545829, FN545832, FN545837, FN545828, FN545835, FN545833, FN545834

HBV/B (n=31)

B1 (n=10): D50521, AB010292, AB073849, AB073852, D23679, D23677, AB073846, D00329, AB073851, AB602818

B2 (n=10): AB073825, AB073831, AF121244, AB073828, AB073821, AB073833, AB073836, AF100309, AB073822, AB073834,

B3 (n=2): M54923, D00331

B4 (n=3): AB031266, AB073835, AY033072

B6 (n=6): DQ463801, DQ463797, AB287314, DQ463799, DQ463787, DQ463795

HBV/C (n=168)

C1 (n=49): AB074756, AF068756, AB112408, AB112348, AB112066, AB112471, AB074047, AB205125, AB112065, AB112472, AB074755, AF223959, AF223956, AF223955, AY217371, AJ748098, AB111946, AB074756, AB112063, AF223954, AY167099, AY217374, AF286594, AP011097, AF223960, AF223957, JQ801478, HM011488, GQ924642, GQ377536, JQ801522, JN827423, KM999990, JQ801486, JN827416, JQ801508, HM011491, GQ924658, JQ801500, GQ924609, JQ429078, GQ924616, JQ801493, KC315399, GQ924612, JQ801470, JQ801517, GQ358153, GQ358154

C2 (n=58): AB205124, AF330110, AY641558, AY641561, AY641563, AY641562, AB205123, AB113876, AB113875, AB113877, AB115417, AY800390, AB033550, X04615, AB033553, D23684, M38636, GQ377535, EU916238, EU939592, GQ377585, AB033556, AY206378, GQ227694, EU939553, EU939536, GQ377514, GQ924633, EU916204, FJ899796, FJ562292, FJ562251, FJ787445, EU939557, KM999991, HM750134, EU589345, EU939647, EU589340, JQ040158, EU939587, FJ386586, X01587, AF533983, AP011098, AB202071, AB113879, AY247031, AB014362, AY057947, EU939630, GQ377539, GQ377556, GQ377604, GQ377631, GQ377549, GQ377613, GQ377590

C3 (n=3): X75656, X75665, AF241410

C4 (n=2): AB048704, AB048705

C5 (n=14): AB241109, AB241113, AP011100, AB241110, EU410081, AP011101, EU410080, JN827414, AB241112, KM999992, AB241111, JN827415, EU410079, AP011099

C6 (n=18): AB493843, AB493840, AB493847, AB493837, AB493842, AB493844, AP011102, AB493838, GQ358156, GQ358155, AB493839, AB493841, AP011103, AB554022, AB554015, AB554021, KM999993, AB554014

C7 (n=2): EU670263, GU721029

C8 (n=6): AP011105, AP011104, AP011107, AP011106, EU306671, EU306672

C9 (n=1): AP011108

C10 (n=1): AB540583

C11 (n=2): AB554019, AB554020

C12 (n=5): AB554025, AB554018, AB560661, GQ358157, AB644285

C13 (n=4): AB644281, AB644282, AB644280, AB644283, AB644284

C14 (n=1): HM011493

C15 (n=1): AB644286

C16 (n=1): AB644287

HBV/D (n=78)

D1 (n=27): AB104709, AB104711, AB104712, AB126581, AB188244, AB222710, AB222711, AB222712, AB222713, AB246347, AB246348, AF121240, AF121241, AF280817, AY161157, AY721605, AY721606, AY721607, AY721608, AY721609, AY721612, AY741797, AY945307, X02496, Y07587, EU594396, EU594397

D2 (n=35): AB078032, AB078033, AB090270, AB109475, AB109476, AB110075, AB116266, AB120308, AB205126, AB205127, AB210822, AB267090, AJ627220, AJ627223, AY090453,

Z35716, EU594403, EU594402, EU594401, EU594400, EU594410, EU594409, EU594408,

EU594407, EU594416, EU594415, EU594428, EU594399, EU594432, EU594431, EU594425, EU594423, EU594422, EU594421, EU594405.

D3 (N=12): AJ131956, AJ344117, AY233291, AY233292, AY233293, AY233294, AY233295, AY233296, DQ111987, EU594382, EU594436, EU594434

D4 (n=3): AB033559, AB048702, AB048703

D5 (n=1): DQ315779

HBV/E (n=37)

AB091255, AB091256, AB201288, AB201290, AB201289, AB201287, X75664, X75657, DQ060822, FN594761, FN594764, AM494711, AM494712, AM494714, AM494706, AM494697, GQ161783, GQ161811, AB205188, FN594749, DQ060823, DQ060825, AY935700, DQ060824, DQ060829, DQ060830, DQ060826, GQ161790, AB205192, AB194948, AM494713,

AB106564, AB194947, FN594752, GQ161828, GQ161791, FN594759

HBV/F (n=38)

F1 (n=14): AY090459, AY090461, AY090458, AY090456, AB116552, AF223964, AY179735, AF223963, HM585199, AB116654, AB086397, EU670262, KJ638656, KJ638663

F2 (n=24): X69798, AY090455, AB116551, AB036920, AB116550, AY179734, AF223962, AB166850, AF223965, AB036915, DQ823090, DQ899148, AY311370, AB036914, AB036919, AB036916, AB036912, AB036911, AB036913, DQ899149, FJ589067, AB036910, X75663, AB116549

HBV/G (n=13)

AB056513, AF405706, AF160501, AB064310, AP007264, DQ207798, EF634481, AB064313, AB064311, AB056514, AB064312, AB056515, AB375170

HBV/H (n=30)

AY090454, AY090457, AY090460, AB059661, HM066946, AB059660, AB375161, AB375163, HM117850, AB516394, HM117851, AB064315, AB516395, AB375159, AB375160, AB516393, AB375162, AB059659, FJ356716, FJ356715, AB375164, AB266536, EU498228, AB353764, AP007261, AB205010, AB298362, EF157291, AB179747, AB275308

HBV/I (n=3)

AF241407, AF241409, AB231908

List of HBV/A with deletion (HBV/A (Del)) in Fig. 5B and 5C

V00866, AF297621, AF297622, AF297625, AF297623, AF297624

List of HBV/C with deletion (HBV/C (Del)) in Fig. 5B and 5C

AB048705, EU306671, EU306672

**List of non-human primate HBVs**

Orangutan (n=16)

Complete genome sequences (n=9)

AF193864, AF193863, EU155825, EU155824, EU155821, EU155822, EU155827, EU155823, EU155826

Partial genome sequences (n=7)

Y17565, Y17564, Y17563, Y17562, Y17561, Y17560, Y17559

Chimpanzee (n=41)

Complete genome sequences (n=27)

AF242586, AF242585, D00220, AF305327, AF222323, AF222322, FJ798099, FJ798098, AB046525, AJ131575, JQ664509, JQ664508, JQ664507, JQ664506, JQ664505, JQ664504, AM117396, AM117395, AY330911, AF242585, AB032433, AB032432, AM117397, AY330912, HQ018764, AF498266, HQ018763

Partial genome sequences (n=14)

FJ798105, FJ798104, FJ798103, FJ798102, FJ798101, FJ798100, AF305330, AF305329, AF305328, AF305326, AJ851166, AJ580975, AJ580974, AJ851168

Gorilla (n=6)

Complete genome sequences (n=6)

JQ664503, JQ664502, FJ798097, FJ798095, FJ798096, AJ131567

Gibbon (n=91)

Complete genome sequences (n=67)

U46935, AB823656, AB823657, AB823658, AB823659, AB823660, AB823661, AB823662, EU155829, EU155828, AJ131568, AY781183, AY781180, AB037928, AJ131572, AJ131571, AJ131569, AY330913, AY330915, AJ131573, AY077735, AJ131574, AY330914, AJ131568, AY077736, AY330917, AY330916, HQ603078, HQ603077, HQ603076, HQ603075, HQ603074, HQ603073, HQ603072, HQ603071, HQ603070, HQ603069, HQ603068, HQ603067, HQ603066, HQ603065, HQ603064, HQ603063, HQ603062, HQ603061, HQ603060, HQ603059, HQ603058, AY781178, AY781177, AY781187, AY781186, AY781185, AY781184, AY781182, AY781181, AY781180, AY781179, KT893897, AJ131570, EU155827, EU155826, EU155825, EU155824, EU155822, EU155821, EU155823

Partial genome sequences (n=24)

AF274499, AF274495, AF477491, AF477490, AF477489, AF477487, AF477494, AF477493, AF477492, AF477488, AF477486, AF477485, AF477484, AF477483, AF477482, AF274496, AF213009, AF213010, AF213008, AF213007, AF213006, AF213005, AF275378, AF275380

**Supplementary Tables**

Supplementary Table S1. Percent identities among Wendy, human HBV/A-I, and non-human primate HBVs in 100 bp of 5’ side of the 6nt insertion specific to HBV/A in the Core region.

|  | A | B | C | D | E | F | G | H | I | Ou | Go | Ch | Gi | WM | Wendy |
| --- | --- | --- | --- | --- | --- | --- | --- | --- | --- | --- | --- | --- | --- | --- | --- |
| A | 98 | 95.3 | 94.7 | 95.7 | 94 | 94.9 | 93 | 91.3 | 94.7 | 93.2 | 94.3 | 95.4 | 94 | 90 | 93 |
| B |  | 96 | 96.4 | 94.3 | 93.7 | 95.2 | 94 | 92.7 | 96.3 | 94.9 | 96.3 | 96.2 | 95.8 | 91 | 94.7 |
| C |  |  | 99.3 | 95.3 | 94.7 | 96 | 94.7 | 93.6 | 98 | 96 | 95 | 97.2 | 98.3 | 89.7 | 97.7 |
| D |  |  |  | 99.3 | 96.7 | 95.3 | 95.7 | 94 | 94.7 | 94 | 94 | 95.7 | 95 | 89 | 95.7 |
| E |  |  |  |  | 100 | 94.7 | 97 | 93.3 | 93.3 | 94.7 | 93.3 | 95 | 95.7 | 90 | 95 |
| F |  |  |  |  |  | 98 | 95.7 | 94 | 94.7 | 94.2 | 95.3 | 96.1 | 95 | 90 | 96.3 |
| G |  |  |  |  |  |  | 100 | 96.3 | 93.3 | 95 | 94.3 | 94.7 | 95.7 | 91 | 95 |
| H |  |  |  |  |  |  |  | 98 | 91.7 | 91.3 | 92.7 | 93.7 | 92.7 | 87.3 | 93.3 |
| I |  |  |  |  |  |  |  |  | 98.7 | 95.2 | 95.7 | 96.3 | 97.7 | 90.3 | 96.3 |
| Ou |  |  |  |  |  |  |  |  |  | 98 | 93.7 | 94.2 | 96.4 | 90.3 | 94.3 |
| Go |  |  |  |  |  |  |  |  |  |  | 99.3 | 96.3 | 94.7 | 91.3 | 93.3 |
| Ch |  |  |  |  |  |  |  |  |  |  |  | 97.3 | 96.2 | 90.3 | 95.3 |
| Gi |  |  |  |  |  |  |  |  |  |  |  |  | 98 | 90.7 | 97.3 |
| WM |  |  |  |  |  |  |  |  |  |  |  |  |  | 100 | 88 |
| Wendy |  |  |  |  |  |  |  |  |  |  |  |  |  |  | 100 |

Numbers are average from three strains each. Numbers in red show the differences of % identities between human HBV/A and Wendy, and GiHBV and Wendy.

A to I, human hepatitis B virus (HBV) genotype A to I; Ou, Orangutan HBV; Go, Gorilla HBV; Ch, Chimpanzee HBV; Gi, Gibbon HBV; WM, Woolly monkey HBV, Wendy, AY330914.

Supplementary Table S2. Percent identities among Wendy, human HBV/A-I, and non-human primate HBVs in 200 bp of 5’ side of the 6nt insertion specific to HBV/A in the Core region.

|  | A | B | C | D | E | F | G | H | I | Ou | Go | Ch | Gi | WM | Wendy |
| --- | --- | --- | --- | --- | --- | --- | --- | --- | --- | --- | --- | --- | --- | --- | --- |
| A | 95.8 | 91.2 | 89.3 | 90.5 | 89.5 | 91.2 | 90.2 | 89.5 | 89.5 | 86.7 | 89.5 | 91.6 | 89.7 | 81.7 | 89.3 |
| B |  | 93.3 | 92.3 | 90 | 89.8 | 91.2 | 91.3 | 89.6 | 92.2 | 89.6 | 90.2 | 91.4 | 91.9 | 82.3 | 92 |
| C |  |  | 98 | 89.4 | 89.8 | 91.6 | 90.2 | 89.9 | 95.7 | 91 | 89.8 | 91.3 | 93.6 | 84.3 | 91.7 |
| D |  |  |  | 98.7 | 95.8 | 89.7 | 90.8 | 86.8 | 89.3 | 88.1 | 87.9 | 89.3 | 89.2 | 81 | 90.3 |
| E |  |  |  |  | 100 | 89.2 | 91.8 | 88.3 | 88.8 | 89 | 89.5 | 90.5 | 90.2 | 82 | 90 |
| F |  |  |  |  |  | 97.3 | 92.3 | 92.5 | 91.9 | 91.4 | 90.7 | 92.3 | 90.4 | 82.3 | 90.5 |
| G |  |  |  |  |  |  | 99.7 | 91 | 90.2 | 89.8 | 90.7 | 92.2 | 92 | 81.3 | 94.3 |
| H |  |  |  |  |  |  |  | 97.3 | 90.5 | 88.2 | 90.3 | 91.3 | 88.9 | 81 | 88.7 |
| I |  |  |  |  |  |  |  |  | 98.7 | 90.7 | 89.7 | 90.9 | 92.9 | 84.7 | 91.7 |
| Ou |  |  |  |  |  |  |  |  |  | 95.5 | 88.7 | 89.3 | 91 | 85.3 | 91.7 |
| Go |  |  |  |  |  |  |  |  |  |  | 99.3 | 95.2 | 90 | 84 | 88.3 |
| Ch |  |  |  |  |  |  |  |  |  |  |  | 96 | 92 | 82.8 | 91.3 |
| Gi |  |  |  |  |  |  |  |  |  |  |  |  | 92.7 | 83.5 | 93.8 |
| WM |  |  |  |  |  |  |  |  |  |  |  |  |  | 100 | 82 |
| Wendy |  |  |  |  |  |  |  |  |  |  |  |  |  |  | 100 |

Numbers are average from three strains each. Numbers in red show the differences of % identities between human HBV/A and Wendy, and GiHBV and Wendy.

A to I, human hepatitis B virus (HBV) genotype A to I; Ou, Orangutan HBV; Go, Gorilla HBV; Ch, Chimpanzee HBV; Gi, Gibbon HBV; WM, Woolly monkey HBV, Wendy, AY330914.

Supplementary Table S3. Percent identities among Wendy, human HBV/A-I, and non-human primate HBVs in 100 bp of 3’ side of the 6nt insertion specific to HBV/A in the Core region.

|  | A | B | C | D | E | F | G | H | I | Ou | Go | Ch | Gi | WM | Wendy |
| --- | --- | --- | --- | --- | --- | --- | --- | --- | --- | --- | --- | --- | --- | --- | --- |
| A | 98.7 | 95.7 | 98.7 | 98 | 94.3 | 91.7 | 91.3 | 92.7 | 97.7 | 92.3 | 92.3 | 93 | 92.3 | 85.6 | 93.3 |
| B |  | 94.7 | 97 | 97 | 92 | 91.3 | 89.3 | 90.8 | 96.7 | 92.7 | 92.7 | 92.9 | 92.2 | 82.8 | 93 |
| C |  |  | 99.3 | 99.3 | 94.3 | 93 | 91.3 | 92.7 | 98.6 | 93.7 | 93.7 | 94.3 | 93.7 | 84.9 | 94.7 |
| D |  |  |  | 99.3 | 93.7 | 93 | 90.7 | 92.3 | 98.3 | 93.7 | 93.7 | 94.3 | 93.7 | 84.5 | 94.7 |
| E |  |  |  |  | 100 | 97.3 | 97 | 98.3 | 95.3 | 98 | 98 | 98.7 | 98 | 86.6 | 99 |
| F |  |  |  |  |  | 98 | 95.7 | 95.7 | 94 | 97.3 | 97.3 | 98 | 97.3 | 85.2 | 98.3 |
| G |  |  |  |  |  |  | 100 | 95.3 | 92.3 | 95 | 95 | 95.7 | 95 | 84.5 | 96 |
| H |  |  |  |  |  |  |  | 99.3 | 93.7 | 97.7 | 97.7 | 97.4 | 96.8 | 85.9 | 97.3 |
| I |  |  |  |  |  |  |  |  | 99.3 | 94.7 | 94.7 | 95.3 | 94.7 | 83.9 | 95.7 |
| Ou |  |  |  |  |  |  |  |  |  | 100 | 100 | 99.3 | 98.7 | 84.5 | 99 |
| Go |  |  |  |  |  |  |  |  |  |  | 100 | 99.3 | 98.7 | 84.5 | 99 |
| Ch |  |  |  |  |  |  |  |  |  |  |  | 99.3 | 98.9 | 85.2 | 99.7 |
| Gi |  |  |  |  |  |  |  |  |  |  |  |  | 98 | 84.5 | 99 |
| WM |  |  |  |  |  |  |  |  |  |  |  |  |  | 100 | 85.6 |
| Wendy |  |  |  |  |  |  |  |  |  |  |  |  |  |  | 100 |

Numbers are average from three strains each. Numbers in red show the differences of % identities between human HBV/A and Wendy, and GiHBV and Wendy.

A to I, human hepatitis B virus (HBV) genotype A to I; Ou, Orangutan HBV; Go, Gorilla HBV; Ch, Chimpanzee HBV; Gi, Gibbon HBV; WM, Woolly monkey HBV, Wendy, AY330914.

Supplementary Table S4. Percent identities among Bassi, human HBV/A-I, and non-human primate HBVs in 100 bp of 5’ side of the polymorphic preS1 region.

|  | A | B | C | D | E | F | G | H | I | Ou | Go | Ch | Gi | WM | Bassi |
| --- | --- | --- | --- | --- | --- | --- | --- | --- | --- | --- | --- | --- | --- | --- | --- |
| A | 96 | 87.8 | 90.6 | 88.4 | 89.5 | 89.9 | 88.2 | 88.9 | 91.2 | 88 | 90.9 | 89.1 | 89.8 | 74.4 | 90 |
| B |  | 96 | 87.2 | 89.9 | 88.2 | 88.1 | 87.5 | 86.5 | 87.5 | 89.1 | 88.9 | 88.2 | 90.5 | 81.5 | 84.2 |
| C |  |  | 97.3 | 91.1 | 88.2 | 89.6 | 86.9 | 88.9 | 93.9 | 91.2 | 91.9 | 89.9 | 90.9 | 78.8 | 88.2 |
| D |  |  |  | 98 | 88.9 | 90.2 | 87.9 | 89.2 | 89.9 | 92.1 | 92.3 | 90.6 | 91.1 | 79.1 | 85.9 |
| E |  |  |  |  | 93.9 | 89.1 | 86.9 | 85.5 | 88.2 | 88 | 90.6 | 89.3 | 87.9 | 79.5 | 88.6 |
| F |  |  |  |  |  | 97.3 | 88.6 | 95 | 91.2 | 90.9 | 95 | 92.9 | 90.1 | 75.8 | 87.5 |
| G |  |  |  |  |  |  | 100 | 85.9 | 88.6 | 88.6 | 87.9 | 86.9 | 85.9 | 78.8 | 84.9 |
| H |  |  |  |  |  |  |  | 100 | 90.6 | 89.9 | 91.9 | 89.9 | 88.9 | 75.8 | 83.8 |
| I |  |  |  |  |  |  |  |  | 99.3 | 90.2 | 92.6 | 90.6 | 91.2 | 77.4 | 87.5 |
| Ou |  |  |  |  |  |  |  |  |  | 96 | 91.2 | 90.6 | 92.3 | 78.1 | 85.9 |
| Go |  |  |  |  |  |  |  |  |  |  | 100 | 97.6 | 92.6 | 78.9 | 89.2 |
| Ch |  |  |  |  |  |  |  |  |  |  |  | 96 | 91.7 | 79.5 | 86.9 |
| Gi |  |  |  |  |  |  |  |  |  |  |  |  | 91.6 | 78.1 | 86.2 |
| WM |  |  |  |  |  |  |  |  |  |  |  |  |  | 100 | 77.8 |
| Bassi |  |  |  |  |  |  |  |  |  |  |  |  |  |  | 100 |

Numbers are average from three strains each. Numbers in red show the differences of % identities between human HBV/E, G and Bassi, and ChHBV and Bassi.

A to I, human hepatitis B virus (HBV) genotype A to I; Ou, Orangutan HBV; Go, Gorilla HBV; Ch, Chimpanzee HBV; Gi, Gibbon HBV; WM, Woolly monkey HBV, Bassi, AB046525.

Supplementary Table S5. Percent identities among Bassi, human HBV/A-I, and non-human primate HBVs in 200 bp of 5’ side of the polymorphic preS1 region.

|  | A | B | C | D | E | F | G | H | I | Ou | Go | Ch | Gi | WM | Bassi |
| --- | --- | --- | --- | --- | --- | --- | --- | --- | --- | --- | --- | --- | --- | --- | --- |
| A | 94.3 | 86.8 | 87.4 | 86.2 | 87.2 | 84.2 | 86.3 | 83.9 | 87.7 | 84.8 | 86.6 | 85.7 | 87.1 | 75.5 | 86.7 |
| B |  | 94.7 | 86.1 | 88.5 | 85.7 | 84.2 | 85.7 | 84.8 | 85.8 | 85.8 | 85.7 | 85.3 | 88.2 | 78.7 | 84 |
| C |  |  | 96.3 | 88.9 | 84.9 | 83.3 | 83.3 | 84.8 | 92.2 | 87.4 | 86.2 | 84.8 | 87.9 | 77 | 86.5 |
| D |  |  |  | 96.7 | 85.7 | 84.4 | 85.8 | 86.7 | 86.6 | 82.9 | 87.3 | 86.4 | 87.7 | 78.6 | 85.7 |
| E |  |  |  |  | 96.3 | 83.6 | 87.2 | 83.5 | 84.2 | 85.9 | 89.8 | 89.4 | 88.2 | 77.7 | 88.2 |
| F |  |  |  |  |  | 91.7 | 82.8 | 87.9 | 84 | 85.2 | 84.3 | 84.9 | 84.3 | 74.7 | 82.7 |
| G |  |  |  |  |  |  | 100 | 82.5 | 83.7 | 84.7 | 85.5 | 85.5 | 84.3 | 76.5 | 84.5 |
| H |  |  |  |  |  |  |  | 98.2 | 85.5 | 85.4 | 85.7 | 85.3 | 85.2 | 75.7 | 83.7 |
| I |  |  |  |  |  |  |  |  | 97.7 | 84.9 | 86.3 | 84.9 | 86.3 | 75.7 | 84.8 |
| Ou |  |  |  |  |  |  |  |  |  | 94.7 | 86.9 | 86.9 | 89.9 | 76.7 | 85.7 |
| Go |  |  |  |  |  |  |  |  |  |  | 99.3 | 93.1 | 89.9 | 77 | 85.8 |
| Ch |  |  |  |  |  |  |  |  |  |  |  | 92.7 | 89.2 | 76.5 | 86.7 |
| Gi |  |  |  |  |  |  |  |  |  |  |  |  | 90.5 | 77.1 | 86 |
| WM |  |  |  |  |  |  |  |  |  |  |  |  |  | 100 | 78 |
| Bassi |  |  |  |  |  |  |  |  |  |  |  |  |  |  | 100 |

Numbers are average from three strains each. Numbers in red show the differences of % identities between human HBV/E, G and Bassi, and ChHBV and Bassi.

A to I, human hepatitis B virus (HBV) genotype A to I; Ou, Orangutan HBV; Go, Gorilla HBV; Ch, Chimpanzee HBV; Gi, Gibbon HBV; WM, Woolly monkey HBV, Bassi, AB046525.

Supplementary Table S6. Percent identities among Bassi, human HBV/A-I, and non-human primate HBVs in 100 bp of 3’ side of the polymorphic preS1 region.

|  | A | B | C | D | E | F | G | H | I | Ou | Go | Ch | Gi | WM | Bassi |
| --- | --- | --- | --- | --- | --- | --- | --- | --- | --- | --- | --- | --- | --- | --- | --- |
| A | 95.3 | 85.1 | 87.4 | 81.7 | 81.1 | 77.4 | 84.3 | 79 | 92.3 | 82.9 | 84.3 | 84.3 | 85.1 | 72 | 83.3 |
| B |  | 94.7 | 81.7 | 83.3 | 80.6 | 76.3 | 79.7 | 79.3 | 86.1 | 81.9 | 86.3 | 85.3 | 85.8 | 72.7 | 85.3 |
| C |  |  | 94.7 | 81.7 | 81.2 | 74.3 | 85 | 80.7 | 89.7 | 81.7 | 82.7 | 83.3 | 85.8 | 72.3 | 82.7 |
| D |  |  |  | 100 | 90.7 | 78.3 | 88 | 84 | 82 | 85 | 87 | 87.3 | 84.3 | 70 | 86 |
| E |  |  |  |  | 98.7 | 76.4 | 86 | 83 | 83.3 | 80.7 | 86.3 | 87.7 | 81.6 | 69.7 | 85.3 |
| F |  |  |  |  |  | 96 | 77 | 93.3 | 77.9 | 77.3 | 78 | 77.4 | 80.1 | 72 | 79 |
| G |  |  |  |  |  |  | 100 | 83 | 87.3 | 83 | 88 | 88 | 83 | 72 | 89 |
| H |  |  |  |  |  |  |  | 100 | 80.3 | 80.3 | 82 | 82.3 | 84.3 | 71 | 83 |
| I |  |  |  |  |  |  |  |  | 99.3 | 85 | 87.3 | 87.3 | 87.7 | 72.7 | 88.3 |
| Ou |  |  |  |  |  |  |  |  |  | 91.3 | 85 | 84.6 | 91.2 | 75 | 84.7 |
| Go |  |  |  |  |  |  |  |  |  |  | 100 | 97 | 88.7 | 68 | 99 |
| Ch |  |  |  |  |  |  |  |  |  |  |  | 95.7 | 88.1 | 69 | 96 |
| Gi |  |  |  |  |  |  |  |  |  |  |  |  | 94.7 | 74.3 | 88.3 |
| WM |  |  |  |  |  |  |  |  |  |  |  |  |  | 100 | 69 |
| Bassi |  |  |  |  |  |  |  |  |  |  |  |  |  |  | 100 |

Numbers are average from three strains each. Numbers in red show the differences of % identities between human HBV/E, G and Bassi, and ChHBV and Bassi.

A to I, human hepatitis B virus (HBV) genotype A to I; Ou, Orangutan HBV; Go, Gorilla HBV; Ch, Chimpanzee HBV; Gi, Gibbon HBV; WM, Woolly monkey HBV, Bassi, AB046525.

Supplementary figure legend

Figure S1. Phylogenetic analysis of hepatitis B virus (HBV) was performed by the neighbor-joining method for human and non-human primate (NHP) HBVs. Human HBV strains and NHP HBV strains showed distinct cluster. HBV/A to HBV/I, HBV genotype A to I; OuHBV, Orangutan HBV; GoHBV, Gorilla HBV; ChHBV, Chimpanzee HBV; GiHBV, Gibbon HBV; WMHBV, Woolly monkey HBV.


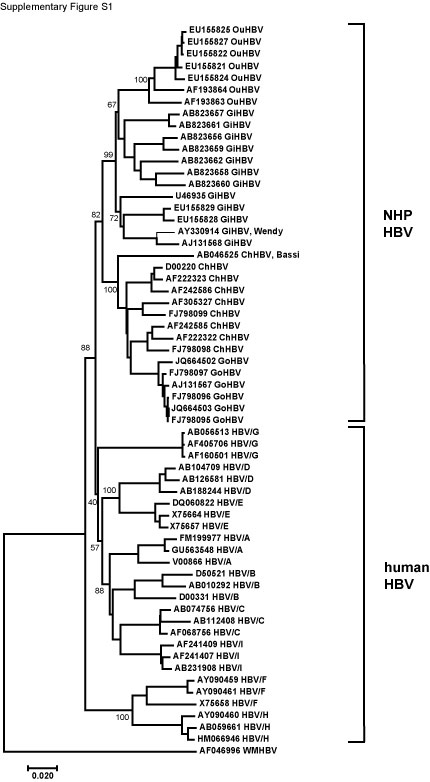

Supplement: Supplementary file 1 — Additional file 1. List of human HBV sequences. List of non-human primate HBVs. Table S1. Percent identities among Wendy, human HBV/A-I, and non-human primate HBVs in 100 bp of 5’ side of the 6nt insertion specific to HBV/A in the Core region. Table S2. Percent identities among Wendy, human HBV/A-I, and non-human primate HBVs in 200 bp of 5’ side of the 6nt insertion specific to HBV/A in the Core region. Table S3. Percent identities among Wendy, human HBV/A-I, and non-human primate HBVs in 100 bp of 3’ side of the 6nt insertion specific to HBV/A in the Core region. Table S4. Percent identities among Bassi, human HBV/A-I, and nonhuman primate HBVs in 100 bp of 5’ side of the polymorphic preS1 region. Table S5. Percent identities among Bassi, human HBV/A-I, and non-human primate HBVs in 200 bp of 5’ side of the polymorphic preS1 region. Table S6. Percent identities among Bassi, human HBV/A-I, and non-human primate HBVs in 100 bp of 3’ side of the polymorphic preS1 region. Figure S1. Phylogenetic analysis of hepatitis B virus (HBV) was performed by the neighbor-joining method for human and non-human primate (NHP) HBVs. [file 12985_2021_1667_MOESM1_ESM.docx]
